# Comparison of strategies for catheter ablation of left posterior fascicular ventricular tachycardia

**DOI:** 10.1093/europace/euad339

**Published:** 2023-11-16

**Authors:** Hui-Qiang Wei, Wanwen Chen, Sini Luo, Zili Liao, Xianhong Fang, Hongtao Liao, Qi Sun, Xiao-Gang Guo, Jian-Du Yang, Jackson J Liang, Shulin Wu, Yumei Xue, Jian Ma, Xianzhang Zhan

**Affiliations:** Department of Cardiology, Guangdong Cardiovascular Institute, Guangdong Provincial People’s Hospital (Guangdong Academy of Medical Sciences), Southern Medical University, No. 102 Zhongshan 2nd Rd, Yuexiu District, 510010 Guangzhou, China; Department of Cardiology, Guangdong Cardiovascular Institute, Guangdong Provincial People’s Hospital (Guangdong Academy of Medical Sciences), Southern Medical University, No. 102 Zhongshan 2nd Rd, Yuexiu District, 510010 Guangzhou, China; Department of Cardiology, Guangdong Cardiovascular Institute, Guangdong Provincial People’s Hospital (Guangdong Academy of Medical Sciences), Southern Medical University, No. 102 Zhongshan 2nd Rd, Yuexiu District, 510010 Guangzhou, China; Department of Cardiology, Guangdong Cardiovascular Institute, Guangdong Provincial People’s Hospital (Guangdong Academy of Medical Sciences), Southern Medical University, No. 102 Zhongshan 2nd Rd, Yuexiu District, 510010 Guangzhou, China; Department of Cardiology, Guangdong Cardiovascular Institute, Guangdong Provincial People’s Hospital (Guangdong Academy of Medical Sciences), Southern Medical University, No. 102 Zhongshan 2nd Rd, Yuexiu District, 510010 Guangzhou, China; Department of Cardiology, Guangdong Cardiovascular Institute, Guangdong Provincial People’s Hospital (Guangdong Academy of Medical Sciences), Southern Medical University, No. 102 Zhongshan 2nd Rd, Yuexiu District, 510010 Guangzhou, China; Arrhythmia Center, National Center for Cardiovascular Diseases, Chinese Academy of Medical Sciences and Peking Union Medical College, Fuwai Hospital, No.167 Beilishi Rd. Xicheng District, 100037 Beijing, China; Arrhythmia Center, National Center for Cardiovascular Diseases, Chinese Academy of Medical Sciences and Peking Union Medical College, Fuwai Hospital, No.167 Beilishi Rd. Xicheng District, 100037 Beijing, China; Arrhythmia Center, National Center for Cardiovascular Diseases, Chinese Academy of Medical Sciences and Peking Union Medical College, Fuwai Hospital, No.167 Beilishi Rd. Xicheng District, 100037 Beijing, China; Division of Cardiovascular Medicine, Cardiac Arrhythmia Service, University of Michigan Health System, Ann Arbor, MI, USA; Department of Cardiology, Guangdong Cardiovascular Institute, Guangdong Provincial People’s Hospital (Guangdong Academy of Medical Sciences), Southern Medical University, No. 102 Zhongshan 2nd Rd, Yuexiu District, 510010 Guangzhou, China; Department of Cardiology, Guangdong Cardiovascular Institute, Guangdong Provincial People’s Hospital (Guangdong Academy of Medical Sciences), Southern Medical University, No. 102 Zhongshan 2nd Rd, Yuexiu District, 510010 Guangzhou, China; Arrhythmia Center, National Center for Cardiovascular Diseases, Chinese Academy of Medical Sciences and Peking Union Medical College, Fuwai Hospital, No.167 Beilishi Rd. Xicheng District, 100037 Beijing, China; Department of Cardiology, Guangdong Cardiovascular Institute, Guangdong Provincial People’s Hospital (Guangdong Academy of Medical Sciences), Southern Medical University, No. 102 Zhongshan 2nd Rd, Yuexiu District, 510010 Guangzhou, China

**Keywords:** Fragmented antegrade Purkinje potential, Catheter ablation, Fascicular ventricular tachycardia, Left posterior fascicular

## Abstract

**Aims:**

Traditional ablation strategies including targeting the earliest Purkinje potential (PP) during left posterior fascicular (LPF) ventricular tachycardia (VT) or linear ablation at the middle segment of LPF during sinus rhythm are commonly used for the treatment of LPF-VT. Catheter ablation for LPF-VT targeting fragmented antegrade Purkinje (FAP) potential during sinus rhythm is a novel approach. We aimed to compare safety and efficacy of different ablation strategies (FAP ablation vs. traditional ablation) for the treatment of LPF-VT.

**Methods and results:**

Consecutive patients with electrocardiographically documented LPF-VT referred for catheter ablation received either FAP ablation approach or traditional ablation approach. Electrophysiological characteristics, procedural complications, and long-term clinical outcome were assessed. A total of 189 consecutive patients who underwent catheter ablation for LPF-VT were included. Fragmented antegrade Purkinje ablation was attempted in 95 patients, and traditional ablation was attempted in 94 patients. Acute ablation success with elimination of LPF-VT was achieved in all patients. Left posterior fascicular block occurred in 11 of 95 (11.6%) patients in the FAP group compared with 75 of 94 (79.8%) patients in the traditional group (*P* < 0.001). Fragmented antegrade Purkinje ablation was associated with significant shorter procedure time (94 ± 26 vs. 117 ± 23 min, *P* = 0.03) and fewer radiofrequency energy applications (4.1 ± 2.4 vs. 6.3 ± 3.5, *P* = 0.003) compared with the traditional group. One complete atrioventricular block and one left bundle branch block were seen in the traditional group. Over mean follow-up of 65 months, 89 (93.7%) patients in the FAP group and 81 (86.2%) patients in the traditional group remained free of recurrent VT off antiarrhythmic drugs (*P* = 0.157).

**Conclusion:**

Left posterior fascicular-ventricular tachycardia ablation utilizing FAP and traditional ablation approaches resulted in similar acute and long-term procedural outcomes. Serious His-Purkinje injury did occur infrequently during traditional ablation. The use of FAP ablation approach was associated with shorter procedure time and fewer radiofrequency energy applications, especially for non-inducible patients.

## Graphical Abstract

**Graphical Abstract:**
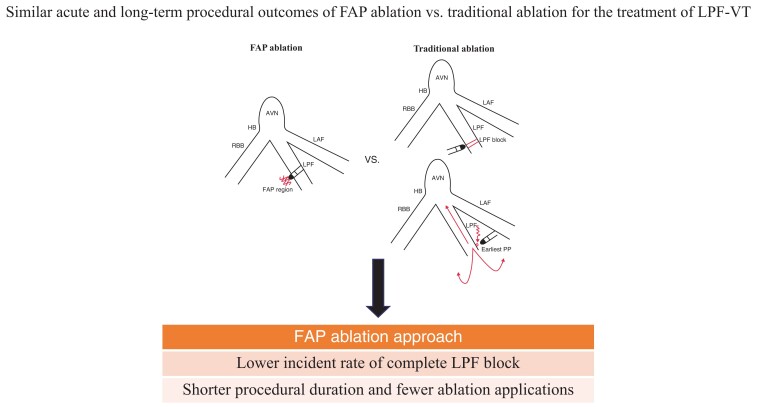

**What’s new?**

- This is the first study to access the long-term efficacy and safety of left posterior fascicular (LPF)-ventricular tachycardia ablation using different ablation approaches.
- Acute and chronic success rate with fragmented antegrade Purkinje (FAP) ablation was comparable with that achieved with traditional ablation.
- No serious His-Purkinje injury was found in FAP ablation, whereas complete atrioventricular block and left bundle branch block were noted in traditional ablation.
- The incident rate of complete LPF block in the FAP ablation was significantly lower than the traditional ablation.
- Compared with traditional ablation, FAP ablation showed significantly shorter procedural duration and fewer ablation applications.

## Introduction

Verapamil-sensitive fascicular ventricular tachycardia (FVT) was first described by Zipes *et al.*^1^ in 1979 with a right bundle branch block (RBBB) and superior left-axis deviation in patients with structurally normal hearts.^1^ Left posterior fascicular (LPF) ventricular tachycardia (VT) is the most common subtype of FVT.^2–4^ Fascicular ventricular tachycardia has been shown to be a re-entrant mechanism involving abnormal Purkinje tissue with decremental properties, left ventricular myocardium, and the left fascicles, though the entire components of the re-entrant circuit remain incompletely defined and understood.^5^ Radiofrequency catheter ablation (RFCA) of LPF-VT is commonly performed using traditional ablation strategies including targeting the earliest PP during LPF-VT^6^ or linear ablation at the middle segment of the LPF during sinus rhythm.^7^ However, several factors, such as non-inducibility or non-sustained nature of LPF-VT and inadvertent LPF block, often pose a difficult challenge in a clinical practice. We previously described a novel approach with favourable outcome targeting fragmented antegrade Purkinje (FAP) potential during sinus rhythm.^8,9^ However, there are little and incomplete data comparing these different ablation approaches for the treatment of LPF-VT. Therefore, we aimed to compare safety and efficacy of different ablation strategies (FAP ablation vs. traditional ablation) for the treatment of LPF-VT.

## Methods

### Study population

**Figure 1:**
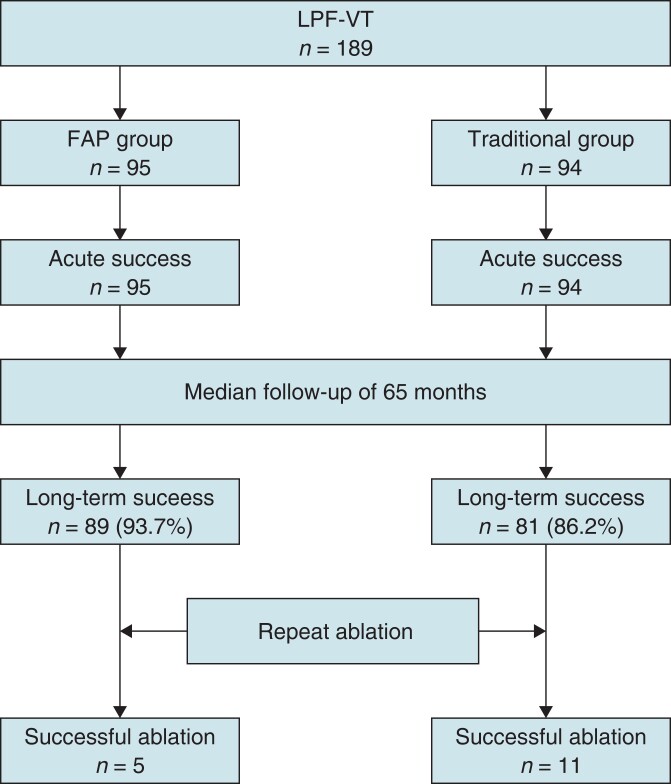
Flowchart of consecutive patients with fascicular ventricular tachycardia enrolled in this study. FAP, fragmented antegrade Purkinje; LPF, left posterior fascicular; VT, ventricular tachycardia.

**Figure 2:**
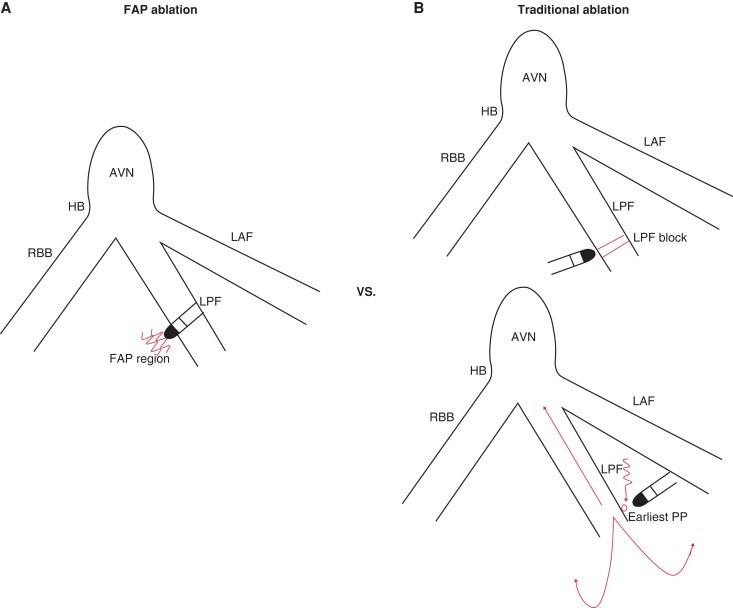
A schematic illustration of FAP and traditional ablation approaches. AVN, atrioventricular node; FAP, fragmented antegrade Purkinje; HB, His bundle; LAF, left anterior fascicular; LPF, left posterior fascicular; PP, Purkinje potential; RBB, right bundle branch.

Patients who underwent index ablation for LPF-VT in Fuwai Hospital and Guangdong Provincial People’s Hospital from January 2012 to December 2019 were consecutively included for present analysis (*Figure 1*). The inclusion criteria were as follows: (i) no structural heart disease with clinical evaluation and cardiac imaging and (ii) documented sustained VT with RBBB morphology and left-axis deviation on electrocardiogram. Patients were divided into two groups based on different ablation approaches: FAP and traditional groups (*Figure 2*). All patients provided written informed consent prior to the catheter ablation. This study was approved by the institutional ethics committee.

### Electrophysiological study

All antiarrhythmic drugs were discontinued for at least five half-lives before the procedure. An electrophysiological study was performed under local anaesthesia. Two quadripolar catheters were inserted from the femoral vein and placed at the His bundle region and right ventricular (RV) apex, and a decapolar catheter was positioned in coronary sinus (CS) via the internal jugular vein or femoral vein approach. Twelve-lead surface electrocardiogram (ECG) and intracardiac electrograms were displayed simultaneously by a digital multichannel system (LabSystem PRO, Bard Electrophysiology, Lowell, MA).The filter setting for bipolar intracardiac electrogram was 30–500 Hz, and unipolar intracardiac electrogram was 0.05–500 Hz.

Stimulation protocol was performed as follows: (i) programmed ventricular stimulation from two sites (RV apex and RV outflow tract) at two drive trains with up to three extrastimuli, (ii) incremental burst pacing from the RV apex and RV outflow tract, and (iii) burst pacing from the CS. If tachycardia could not be induced in the baseline state, burst pacing and programmed ventricular stimulation were repeated following infusion of isoproterenol (1–3 µg/min).

A 4 mm non-irrigated catheter (Biosense Webster Inc., Diamond Bar, CA, USA) or 3.5 mm irrigated catheter (NaviStar Thermo-Cool, Biosense Webster Inc., Diamond Bar, CA, USA) was introduced into the left ventricle (LV) via retrograde aortic approach. 3D electroanatomic mapping was performed using the CARTO mapping system (Biosense Webster, Inc., Diamond Bar, CA). Intravenous heparin was administered to maintain an activated clotting time of >250 s. The FAP ablation approach or traditional ablation approach was performed dependent on the operator preference.

### Fragmented antegrade Purkinje ablation procedure

**Figure 3:**
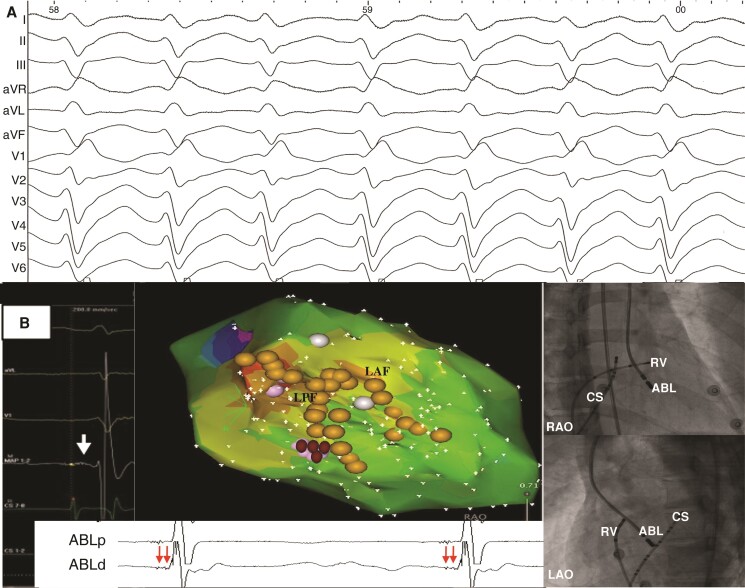
Successful ablation targeting FAPs during sinus rhythm. (*A*) Twelve-lead surface ECG recordings via the electrophysiology recording system during ventricular tachycardia. (*B*) Left: Note that an area with FAP was located within the posterior Purkinje fibre network, and successful ablation was achieved by targeting FAPs. Right: The radiographic images of RAO 30° and LAO 45° showed the catheter positions. ABL, ablation catheter; CS, coronary sinus; FAP, fragmented antegrade Purkinje potential; LAF, left anterior fascicle; LAO, left anterior oblique; LPF, left posterior fascicle; RAO, right anterior oblique; RV, right ventricle.

Detailed point-by-point LV mapping was performed during sinus rhythm, and the left His-Purkinje system including LPF and left anterior fascicle (LAF) was reconstructed by mapping the antegrade Purkinje potentials (PPs). Then, further detailed mapping of the Purkinje fibre network was concentrated in the vicinity of the LPF during sinus rhythm. We have previously described ‘FAP’ as abnormal, wide, fragmented, and low-frequency potentials which precede ventricular activation during sinus rhythm.^8^ Radiofrequency current was delivered at the FAP region during sinus rhythm with temperature-controlled mode at 43°C and irrigation rate at 17 mL/min (*Figure 3*). Energy output of 30–35 W was applied and maintained for 30–60 s at each ablation site.^10^ The endpoint of ablation was defined as non-inducibility of VT after ablation despite programmed stimulation and isoproterenol infusion and elimination of the FAPs during sinus rhythm.

### Traditional ablation procedure

**Figure 4:**
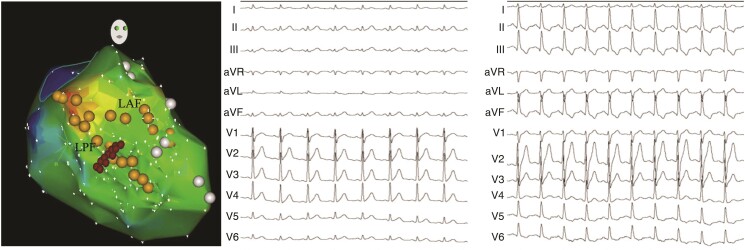
Radiofrequency catheter ablation of LPF-VT with the development of LPF block in a patient with non-inducible VT. (*A*) Three-dimensional electroanatomical map. (*B*) Normal ECG prior to ablation. (*C*) Electrocardiogram manifestation of left posterior fascicular block after ablation. LAF, left anterior fascicle; LPF, left posterior fascicle; VT, ventricular tachycardia.

Activation mapping during LPF-VT was performed, and site with the earliest PP was identified. If the LPF-VT could not be induced or sustained, linear ablation at the middle segment of the LPF during sinus rhythm was performed and achievement of LPF block was identified (*Figure 4*). Left posterior fascicular block was defined as the frontal axis between 90° and 180°, rS pattern in leads I and aVL, qR pattern in leads III and aVF, and QRS duration of <120 ms.^11^ The ablation was considered successful if LPF-VT was terminated during ablation and the patient was rendered non-inducible for after ablation despite programmed stimulation and isoproterenol infusion.

### Follow-up

After the procedure, patients remained on continuous telemetric monitoring for >24 h. No antiarrhythmic drugs were prescribed after ablation procedure. After hospital discharge, patients were seen in follow-up in outpatient clinic or virtually (via telephone) every 3 months for the first year post-ablation and every 6 months thereafter. All patients underwent a 12-lead ECG or 24 h Holter monitoring during follow-up. If the patients had any rhythm-related symptoms, additional 12-lead ECG or 24 h 12-lead Holter monitoring was performed to document the cause of the symptoms.

### Statistical analysis

All continuous variables are described by mean ± standard deviations. The categorical variables are expressed as numbers and percentages. Categorical variables were compared using χ^2^ analysis. Continuous variables were compared using the Student’s *t*-test or Mann–Whitney *U* test, depending on data distribution. The difference in freedom from VT recurrence was compared by means of Kaplan–Meier analysis with log-rank test. A two-tailed value of *P* < 0.05 was considered statistically significant. All statistical analyses were performed using SPSS 26.0 (SPSS, Inc., Chicago, IL, USA).

## Results

### Baseline characteristics

A total of 189 consecutive patients who underwent catheter ablation for LPF-VT were included. Fragmented antegrade Purkinje ablation was performed in 95 patients, and traditional ablation was performed in 94 patients. All patients had no structural heart disease. Baseline characteristics of patients are presented in *Table 1*.

**Table 1:** Baseline clinical characteristics between two groups

	FAP group	Traditional group	*P* value
	(*n* = 95)	(*n* = 94)	
Male gender (%)	80 (84.2)	81 (86.2)	0.892
Age (years)	28.1 ± 10.5	30.2 ± 12.1	0.241
Hypertension (%)	10 (10.5)	8 (8.5)	0.637
AADs prior ablation	46 (48.4)	40 (42.6)	0.418
No. of failed AADs	1.6 ± 1.1	1.5 ± 1.2	0.453
LVDd (mm)	42.2 ± 6.2	41.8 ± 4.3	0.482
LVEF (%)	62.9 ± 5.9	64.2 ± 6.2	0.376

Values are expressed as mean ± SD or as *n* (%).

AAD, antiarrhythmic drug; LVDd, left ventricular diastolic diameter; LVEF, left ventricular ejection fraction.

### Electrophysiological study

During electrophysiological study, clinical LPF-VT was induced in 86 of 95 (90.5%) patients in the FAP group and 80 of 94 (85.1%) patients in the traditional group. All inducible VTs demonstrated RBBB morphology and left-axis deviation. Concurrent supraventricular tachycardia was observed in two patients in the FAP group and one patient in the traditional group. There were no significant differences in QRS duration during VT (134.2 ± 47.1 vs. 130.2 ± 34.7 ms, *P* = 0.542), HV interval during VT (−17.1 ± 7.0 vs. −18.4 ± 5.2 ms, *P* = 0.533), or mean tachycardia cycle length (340.4 ± 65.8 vs. 353.4 ± 51.2 ms, *P* = 0.165) between the FAP and traditional groups. There was no difference in used catheters between the two groups.

### Radiofrequency catheter ablation

In the FAP group, catheter ablation was performed targeting the FAP region during sinus rhythm in 92 of 95 (94.7%) patients, while in the 3 patients in whom FAP was not identified, ablation targeting the earliest PP was successful. A median of 4 (2–8) RFCA applications were applied. Left posterior fascicular block occurred in 11 of 95 (11.6%) patients after ablation, whereas 22 (23.2%) patients exhibited rightward shift in frontal axis compared with baseline. The mean procedural duration in the FAP group was 94 ± 26 min. In the traditional group, catheter ablation was performed either during VT in 41 of 94 (43.6%) patients, which targeted the earliest PPs or during sinus rhythm in 53 of 94 (56.4%) patients, which targeted the high-frequency sharp PPs along posterior mid-septal LV using a median of 6 (3–15) RFCA applications. Time to termination for those patients with sustained VT was 8 ± 3 s. The development of new onset LPF block was observed in 75 of 94 (79.8%) patients. Ablation was acutely successful in all patients. The average procedural duration in the traditional group was 117 ± 23 min (*Table 2*).

**Table 2:** Electrophysiologic characteristics and ablation data between FAP and traditional groups

	FAP group	Traditional group	*P* value
	(*n* = 95)	(*n* = 94)	
Inducible rate of VT	86 (90.5)	80 (85.1)	0.254
Concurrent SVT	2 (2.1%)	1 (1.1%)	0.567
QRS duration during VT (ms)	134.2 ± 47.1	130.2 ± 34.7	0.542
Tachycardia cycle length (ms)	340.4 ± 65.8	353.4 ± 51.2	0.165
Complete LPF block	11 (11.6%)	75 (79.8%)	<0.001
Serious His-Purkinje injury	0 (0%)	2 (2.1%)	0.153
Total no. of RFCA application	4.1 ± 2.4	6.3 ± 3.5	0.003
Procedural duration (minutes)	94 ± 26	117 ± 23	0.03

Values are expressed as mean ± SD or as *n* (%).

FAP, fragmented antegrade Purkinje; LPF, left posterior fascicle; RFCA, radiofrequency catheter ablation; SVT, supraventricular tachycardia; VT, ventricular tachycardia.

### Procedural complications

No major complications were observed. Peripheral vascular complications occurred in two patients (one pseudoaneurysm and one arteriovenous fistula) in the FAP group and four (two pseudoaneurysm and two arteriovenous fistula) in the traditional group (*P* = 0.399). Serious His-Purkinje injury occurred in two patients in the traditional group: one patient developed permanent complete atrioventricular block requiring pacemaker implant, and one patient experienced left bundle branch block. No significant conduction system injury was noted in the FAP group.

### Long-term clinical outcome

**Figure 5:**
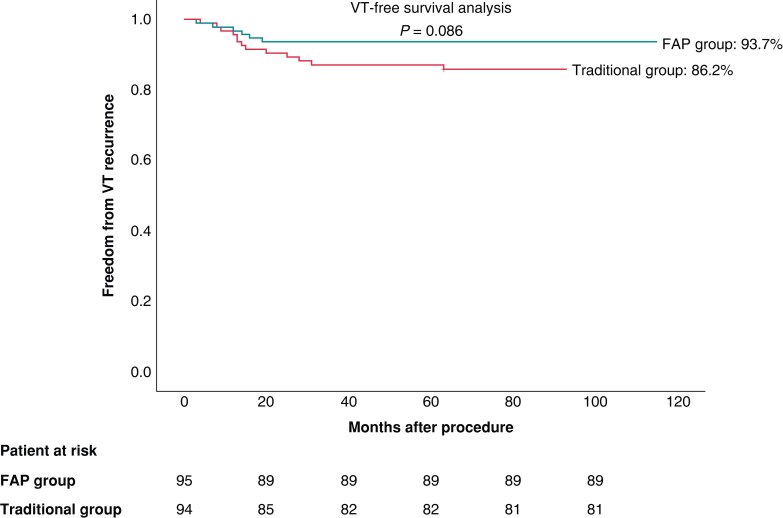
Kaplan–Meier analysis of long-term freedom from LPF-VT recurrence after the procedure. FAP, fragmented antegrade Purkinje; VT, ventricular tachycardia.

Over a mean follow-up period of 65 (13–114) months, 89 (93.7%) patients in the FAP group and 81 (86.2%) patients in the traditional group remained free of recurrent VT off antiarrhythmic drugs (*P* = 0.086; *Figure 5*). Of the 19 patients who had VT recurrence, the recurrent VT had ECG morphology similar to the clinical LPF-VT targeted at the index ablation in 5 of 6 (83.3%) patients in the FAP group and 9 of 13 (69.2%) patients in the traditional group. All consented to repeat ablation. The VT induced during the repeat procedure had similar electrophysiological parameters. Interestingly, in the remaining five patients, recurrent VT exhibited a different ECG morphology with narrower QRS duration. One patient in the FAP group declined second ablation, and 2 of 4 patients in the traditional group agreed to undergo repeat ablation. During the second ablation procedure, upper septal VT and bundle branch re-entry VT in the two patients were diagnosed and repeat ablation was successful in these two patients.

## Discussion

### Major findings

To our knowledge, this is the first study to access the long-term efficacy and safety of LPF-VT ablation using different ablation approaches. In this large case series of LPF-VT ablation, we comprehensively compared FAP ablation with traditional ablation in terms of electrophysiological characteristics, procedural complications, and long-term clinical outcome. We found that (i) acute and chronic success rate with FAP ablation was comparable with that achieved with traditional ablation; (ii) no serious His-Purkinje injury was found in FAP ablation, whereas complete atrioventricular block and left bundle branch block were noted in traditional ablation; (iii) the incident rate of complete LPF block in the FAP ablation was significantly lower than the traditional ablation; and (iv) compared with traditional ablation, FAP ablation showed significantly shorter procedural duration and fewer ablation applications.

### Electrophysiological mapping and ablation characteristics

In this study, though there were no significant differences between the FAP and traditional groups with regard to the acute and chronic success rate, the incident rate of complete LPF block in the FAP group was significantly lower compared with the traditional group (11.6% vs. 79.8%, *P* < 0.001). Furthermore, in the FAP group, FAP could not be mapped in three patients and traditional technique was performed. This new method has about 5% crossover to the traditional technique. Significantly shorter procedural and radiofrequency energy duration and fewer ablation applications were observed in the FAP group. Shorter procedural duration of FAP ablation procedure was likely due in part to the avoidance of repeated aggressive and lengthy stimulation protocols used in the traditional group in order to induce VT. No serious His-Purkinje injury was found in the FAP group, while serious His-Purkinje injury was recorded in 2.1% of patients in the traditional group. Some serious His-Purkinje injuries may be caused by sudden catheter movement following VT termination during ablation. Our encouraging findings therefore suggest that catheter ablation of LPF-VT targeting FAPs during sinus rhythm is safe and feasible and can minimize iatrogenic LPF block during ablation.

### Catheter ablation strategy for left posterior fascicular-ventricular tachycardia

Traditional ablation approaches for LPF-VT with successful outcomes have been previously described including targeting of the earliest PP^6,10,12,13^ or linear ablation at the middle segment of the LPF during sinus rhythm.^7,14^ Liu *et al.*^6^ reported 120 patients undergoing LPF-VT ablation by targeting the earliest PP. They found that 20% of patients developed new-onset LPF block and 58.3% of patients exhibited rightward shift in frontal axis. During a mean follow-up of 55.7 months, freedom from recurrent VT was 80% of patients. Luo *et al.*^7^ studied a total of 195 LPF-VT patients who had undergone catheter ablation with the development of LPF block. With a mean of follow-up of 85 months, 86.9% of patients had long-term freedom from VT. However, the limitations of these approaches are obvious, such as non-inducibility or non-sustained nature of LPF-VT and the development of inadvertent LPF block, which raises the question of whether an alternative targeted approach of ablation during sinus rhythm can be similarly effective without causing iatrogenic LPF block. We have previously described a novel substrate-based approach to ablation of LPV-VT during sinus rhythm.^8^ We have shown that FAPs were likely to represent the slow conduction regions which may represent critical sites for re-entry.^9^ Excellent long-term outcome targeting the FAPs in sinus rhythm was achieved. In this study, ablation targeting the FAP region was performed in 95 patients and 93.7% of patients remained free of recurrent VT during a mean follow-up period of 65 months. Therefore, our results demonstrated that this may be a promising approach alternative to traditional ablation approach.

### Potential advantages of fragmented antegrade Purkinje ablation over traditional ablation

When LPF-VT is not able to be induced during the procedure, ablation success may be limited due to the absence of a reliable procedural endpoint. As such, the FAP ablation approach used in this study may provide several advantages. Firstly, using non-inducibility as the procedural endpoint may be confounded by the fact that mechanical termination may frequently occur during activation mapping. Elimination of FAPs during sinus rhythm may be a simpler and more objective endpoint to confirm successful ablation. Secondly, this approach may minimize the risk of causing iatrogenic LPF block (in a cohort of patients who are typically young and otherwise healthy), which was actually used as an acceptable procedural endpoint during delivery of linear ablation in prior studies^7,15,16^ Thirdly, procedural duration can be shortened obviating the need for aggressive and lengthy stimulation protocols to induce VT. Fourthly, fewer radiofrequency energy applications are required which may reduce the risk atrioventricular nodal or bundle bunch injury and proarrhythmia.

### Study limitations

There are several limitations in the present study. This was a retrospective study. A prospective randomized controlled trial is necessary to confirm our findings. Furthermore, multielectrode catheters (i.e. HD Grid, Optrell, PentaRay, and OctaRay) were not used in our study, which may provide more high-fidelity mapping and visualization of FAPs and PPs while minimizing mechanical termination compared with point-by-point ablation catheter mapping.

## Conclusion

Left posterior fascicular-ventricular tachycardia ablation utilizing FAP ablation and traditional ablation approaches resulted in similar acute and long-term procedural outcomes. Serious His-Purkinje injury did occur infrequently during traditional ablation. The use of FAP ablation approach was associated with shorter procedure time and fewer radiofrequency energy applications, especially for non-inducible patients. The lower rate of complete LPF block might be different if high-density mapping catheters were used.

## Data Availability

The data that support the findings of this study are available from the corresponding author upon reasonable request.
